# Frontal EEG asymmetry in borderline personality disorder is associated with alexithymia

**DOI:** 10.1186/s40479-017-0071-7

**Published:** 2017-09-29

**Authors:** Vera Flasbeck, Stoyan Popkirov, Martin Brüne

**Affiliations:** 1 LWL University Hospital Bochum, Department of Psychiatry, Psychotherapy and Preventive Medicine, Division of Cognitive Neuropsychiatry and Psychiatric Preventive Medicine, Ruhr-University, Alexandrinenstr 1, 44791 Bochum, Germany; 2 Department of Neurology, University Hospital Knappschaftskrankenhaus Bochum, Ruhr-University Bochum, Bochum, Germany

**Keywords:** EEG asymmetry, Borderline personality disorder, Alexithymia

## Abstract

**Background:**

Frontal EEG asymmetry is a widely studied correlate of emotion processing and psychopathology. Recent research suggests that frontal EEG asymmetry during resting state is related to approach/withdrawal motivation and is also found in affective disorders such as major depressive disorder. Patients with borderline personality disorder (BPD) show aberrant behavior in relation to both approach and withdrawal motivation, which may arguably be associated with their difficulties in emotion processing. The occurrence and significance of frontal EEG asymmetry in BPD, however, has received little attention.

**Results:**

Thirty-seven BPD patients and 39 controls underwent resting EEG and completed several psychometric questionnaires. While there were no between-group differences in frontal EEG asymmetry, in BPD frontal EEG asymmetry scores correlated significantly with alexithymia. That is, higher alexithymia scores were associated with relatively lower right-frontal activity. A subsequent analysis corroborated the significant interaction between frontal EEG asymmetry and alexithymia, which was moderated by group.

**Conclusions:**

Our findings reveal that lower right frontal EEG asymmetry is associated with alexithymia in patients with BPD. This finding is in accordance with neurophysiological models of alexithymia that implicate a right hemisphere impairment in emotion processing, and could suggest frontal EEG asymmetry as a potential biomarker of relevant psychopathology in these patients.

## Introduction

Emotion dysregulation is a central feature of borderline personality disorder (BPD) and is often closely related to impairments in emotional awareness [1]. Conceptualized as alexithymia, this difficulty to recognize and describe one’s own emotions has been found to be a crucial mediator of BPD pathology [2] and is predictive of psychotherapy outcome [3]. The neurobiological underpinnings of alexithymia are still poorly understood [4], so identifying a neurophysiological signature or marker to complement self-report instruments would be useful for research and clinical practice.

In electrophysiological studies of emotion processing, local electroencephalographic (EEG) activity in the alpha range – a correlate of relative neural inactivity – has been found to be lateralized over the frontal brain regions in association with certain emotional modalities and various affective disorders [5]. This so-called frontal EEG asymmetry has been explored as a biomarker in mood and anxiety disorders [6–9], as well as posttraumatic stress disorder [10]. With regard to depression, for example, studies reported relatively greater right-frontal resting activity [11], while elevated relative left-frontal EEG activity in patients with cyclothymia or bipolar II disorder predicted conversion to bipolar I disorder [9]. Another line of research posits that frontal EEG asymmetry is related to motivational factors, with greater right frontal activity being a marker of withdrawal and left-frontal activity being associated with approach [11]. These observations (see [5] and [11] for reviews of this topic) suggest that frontal EEG asymmetry could potentially be utilized as a marker of pathology in borderline personality disorder (BPD). However, only one study has so far investigated frontal EEG asymmetry in BPD, in which affective symptoms are highly prevalent and emotional dysregulation is a key feature [12]. Beeney and colleagues reported greater left frontal activity in patients with BPD following rejection but no asymmetry at rest [12]. Patients with major depressive disorder showed greater right frontal activity after rejection consistent with withdrawal behavior. Even though depression, approach and avoidance behavior are common in BPD, their impact on frontal EEG asymmetry in BPD has not been studied. Nor is it entirely clear how alexithymia may contribute to frontal EEG asymmetry in BPD [13, 14]. This could be worth studying though, because Imperatori et al. recently reported an association between alexithymia and EEG power spectra and connectivity during resting state in the default-mode network with lower alpha power in the right posterior cingulate cortex and decreased alpha connectivity in non-clinical alexithymic subjects compared to non-alexithymic subjects [15]. In addition, frontal asymmetry has been proposed as an electrophysiological correlate of a functional gateway, which regulates behavioral responses to emotional stimuli by modulating emotional reactivity [16]. Thus, resting frontal EEG may be a valuable measure to investigate mechanisms involved in emotion processing, including ones related to dysfunctions in conditions such as BPD. Therefore, the aim of the current study was to explore frontal asymmetry of resting-state EEG in patients with BPD, and whether it correlates with alexithymia or other psychopathological measures.

## Methods

### Participants

Thirty-seven patients diagnosed with BPD by an experienced psychiatrist with expertise in BPD according to criteria of the Diagnostic and Statistical Manual of Mental Disorders, 5th edition, were recruited from the LWL-University Hospital Bochum, and 39 healthy control participants (HC) were recruited via advertisement. Only women aged between 18 to 50 were included. Exclusion criteria were neurological illness and, for healthy controls, any known psychiatric disease (self-report). Table 1 shows comorbid disorders, medication and treatment of the patient group.

**Table 1:** Comorbid disorders, medication and the cause of inpatient stay of patients with BPD

	N	%
Comorbid disorders of BPD patients		
Depressive episode	17	43.6
Posttraumatic Stress Disorder	7	18,0
Phobic Disorder	2	5.1
Eating Disorder	5	12.8
Cannabis misuse	7	18.0
Alcohol misuse	11	28.2
Other substance misuse	4	10.3
Medication		
without regular medication	15	38.5
antidepressant	15	38.,5
antidepressant and antipsychotic drugs	9	23.1
additional anticonvulsiva	2	5.1
additional other hypnotic drugs	2	5.1
Reason for inpatient stay		
Dialectical behavior therapy	29	74.4
crisis intervention	10	25.6

The frequency is described by absolute (N) and relative (%) amounts

### EEG recording

Resting EEG was recorded over 4 min from 32 scalp electrodes arranged according to the 10–20 system by BrainVision Recorder (Brain Products GmbH) with impedances kept below 5 kΩ as described previously [17]. Patients were asked to keep their eyes closed. For data analysis (BrainVision Analyzer, Brain Products GmbH) 50 Hz-notch and band pass filters (0.1–100 Hz) were applied; eye movement and muscle artifacts were removed manually and using Independent Component Analysis. Computer-averaged mastoid channels were used for referencing. Four 1-min data blocks were segmented into 2-s-epochs that overlap by 1.5 s. Artifact-free epochs were extracted using a Hamming windows and underwent fast Fourier transformation. Frontal EEG asymmetry scores (FAS) were calculated as the difference of natural log transformed alpha power (8–13 Hz) over F8 and F7 and F4 and F3 (ln[Right]-ln[Left]); higher FAS would thus imply relatively greater right alpha and hence relatively greater left neural activity [1]. FAS was calculated for each 1-min data block and then averaged for all 4 blocks.

### Questionnaires

Participants completed the German version of the Toronto Alexithymia scale (TAS-20; [18]) to assess alexithymia; the Mehrfachwahl–Wortschatz–Intelligenz Test, version B (MWT-B; [19]) to estimate IQ; the Symptom Checklist of Derogatis (SCL-90-R; [20]) to assess general psychopathology; and the Beck Depression Inventory II (BDI-II; [21]) to quantify depressive symptoms. T-Test for independent samples was you used to compare metrics between groups with significance level set at *p * < 0.05. Spearman’s rank correlation coefficient (*r*) was calculated to quantify associations between psychometric measures and FAS. Bonferroni-Holm correction for multiple comparisons was applied for correlation analysis with FAS and questionnaires for each questionnaire separately. For investigation of a moderating effect of group we used the macro tool PROCESS developed by Hayes [22]. The moderation analyses was conducted for TAS total score as the independent variable (X), FAS as the outcome variable (Y) and group (BPD vs. HC) as the mediator (M).

## Results

BPD patients (*n* = 37) and healthy controls (HC; *n* = 39) did not differ significantly in mean age (BPD 26.8 *SD* = 7.5 vs. HC 23.7 *SD* = 5.8 years) and IQ (BPD 103 *SD* = 16.8 vs. HC 110 *SD* = 16.4). Patients had significantly higher ratings of alexithymia with regard to the total score (TAS-20 total score BPD 61.8 *SD* = 11.7 vs. HC 40.6 *SD* = 9.5, *t*_74_ = 8.68, *p* < 0.001) and all subscales (TAS-Difficulties identifying feeling BPD 24.2 *SD* = 4.5 vs. HC 13.2 *SD* = 4.2, *t*_74_ = 11.08, *p* < 0.001; TAS-Difficulties describing feeling BPD 17.2 *SD* = 4.6 vs. HC 11.0 *SD* = 4.0, *t*_74_ = 6.19, *p* < 0.001; TAS-Externally oriented thinking BPD 20.5 *SD* = 5.6 vs. HC 16.5 *SD* = 4.0, *t*_74_ = 3.66, *p* = 0.001). Patients with BPD reported more depressive symptoms as assessed by BDI-II (BPD 38.5 *SD* = 9.1 vs. HC 6.2 *SD* = 6.6, *t*_73_ = 17.56, *p* < 0.001) and more severe current psychopathology as self-reported in the SCL-90-R (GSI: BPD 79.8 *SD* = 1.4 vs HC 54.7 SD = 16.7, *t*_71_ = 9.33, *p* < 0.001; PST: BPD 76.7 *SD* = 4.1 vs HC 49.9 *SD* = 16.7, *t*_70_ = 7.80, *p* < 0.001; PSDI: BPD 125.2 *SD* = 25.7 vs HC 56.5 *SD* = 9.8, *t*_71_ = 14.68, *p* < 0.001).

**Fig. 1:**
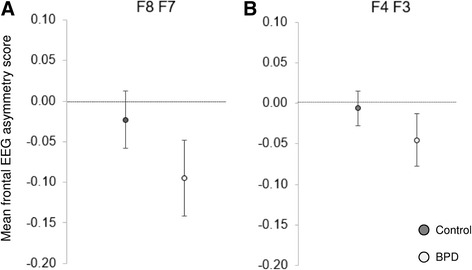
Mean frontal EEG asymmetry scores for healthy subjects and borderline personality disorder patients for F8-F7 (**a**) F4-F4 electrodes (**b**) . Error bars denote standard error of the mean

**Fig. 2:**
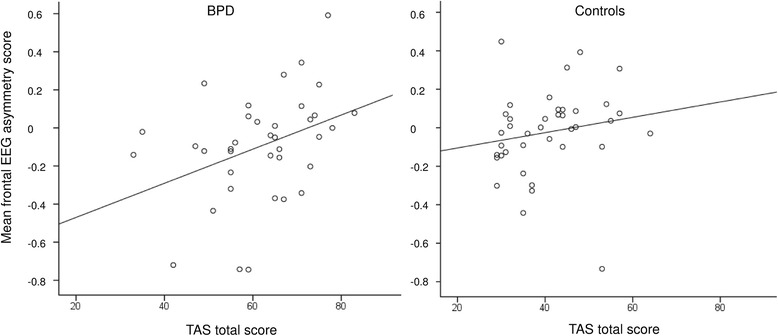
Mean frontal EEG asymmetry scores for patients with borderline personality disorder (left) and healthy control participants (right). Scatter Plots show the relation of the TAS total scores and FAS scores over F8-F7

Average FAS did not differ significantly between patients and healthy controls (F8-F7 BPD-0.095 *SD* = 0.28 vs. HC -0.023 *SD* = 0.22, *t*_74_ = −1.24, *p* = 0.220; F4-F3 BPD-0.046 *SD* = 0.20 vs. HC -0.006 *SD* = 0.13, *t*_74_ = −1.03, *p* = 0.308). Figure 1 A and B visualize FAS in patients with BPD and healthy controls for both electrode pairs. Calculation of Spearman correlation coefficients for psychometric measures with FAS of F8-F7 in the BPD group showed a correlation with the TAS-20 total score that remained significant after Bonferroni-Holm correction (*r* = 0.41; *p* = 0.013; Bonferroni-Holm correction *p* = 0.013). Correlations with other psychometric measures within the BPD and HC groups are shown in Table 2. In contrast, TAS-scores were low in the control group and no correlation with TAS survived correction. In support of the association of FAS and TAS in patients with BPD, a analysis showed a significant interaction of alexithymia with frontal asymmetry moderated by group (overall model: *F* (3, 72) = 2.71, *p* = 0.051, *R* ^*2*^ = 0.10; Interaction *b* = −0.01, *t*(72) = −2.10, *p* = 0.039). Figure 2 depicts the correlations in BPD and the floor effect in controls. Correlations of BDI-II and the subscales of SCL-90-R with FAS were not significant.

**Table 2:** Correlations of FAS of F8-F7 and F4-F3 with psychometric measures in patients with BPD and healthy controls (HC)

Correlation of FAS		BPD		HC	
With variable		*r*	*p*	*r*	*p*
F8-F7	TAS DIF	0.310	0.062	0.258	0.113
	TAS DDF	0.401	0.014	0.307	0.057
	TAS EOT	0.227	0.177	0.205	0.211
	TAS total score	**0.406**	**0.013** ^**a**^	0.362	0.023
	BDI	0.064	0.712	0.203	0.227
	SCl GSI	0.222	0.207	0.341	0.034
	SCL PST	0.088	0.628	0.235	0.150
	SCL PSDI	0.276	0.114	0.318	0.049
F4-F3	TAS DIF	0.193	0.252	−0.144	0.381
	TAS DDF	0.162	0.339	0.069	0.674
	TAS EOT	0.145	0.392	0.204	0.213
	TAS total score	0.236	0.159	0.082	0.620
	BDI	−0.042	0.802	0.044	0.797
	SCl GSI	0.151	0.395	−0.112	0.497
	SCL PST	−0.103	0.567	−0.055	0.741
	SCL PSDI	0.253	0.149	0.020	0.903

The correlation of FAS F8-F7 with TAS total score in patients with BPD is the only correlation surviving Bonferroni-Holm correction (marked with^a^ and bold writing)

### Discussion

The aim of this study was to explore frontal EEG asymmetry in BPD and its relation to alexithymia and depression. Patients did not differ in frontal EEG activity from healthy controls. This finding is in line with the study by Beeney and colleagues, who also found no frontal EEG asymmetry at rest [12]. Notably, correlation analyses did not reveal significant associations with the SCL-90-R or the BDI-II (even though scores were pathologically elevated in BPD), as previous reports in patients with affective disorders would have suggested [6]. This lack of an association could suggest that frontal EEG asymmetry in BPD potentially caused by the comorbid depression may be overridden by other factors. One possible explanation could be that depressivity in BPD differed from the one found in major depression disorder (MDD), even though depression ratings as for example the BDI are comparable high [23, 24]. Thus, the influence of depression on FAS may be distinct in MDD and BPD.

Interestingly, the frontal EEG asymmetry as quantified by FAS correlated significantly with alexithymia in the patient group. Specifically, our additional analysis showed that the interaction of FAS and TAS was moderated by group. Together, this suggests a relatively lower right-frontal activity in BPD patients with high alexithymia scores. In line with the literature on motivational responses (avoidance vs. approach; [5]), this would imply that low-alexithymic patients with BPD would show a tendency for avoidance and withdrawal, while those with high alexithymia measures would be predisposed for approach-oriented actions. Such a conceptual dichotomization of personality structure in BPD has been proposed previously for adolescent patients [25], and it would be plausible to consider alexithymia as an underlying factor.

Our finding is also consistent with neurophysiological models of alexithymia implying alterations in interhemispheric transfer of emotional information and a right hemisphere impairment in emotion processing [13, 26–28]. For example, patients with right hemispheric lesions were found to be more alexithymic than those with left-sided lesions [29].

A limitation of our study is that the participants’ current emotional state was not controlled for, which is known to influence frontal EEG asymmetry [5], and which may be specifically relevant for psychiatric conditions characterized by rapid mood swings such as BPD [30]. While the use of self-report measures is another considerable limitation of the present study, our findings suggest that FAS may potentially be utilized as a biomarker for psychopathological features such as alexithymia in BPD. This is especially relevant, since alexithymia seems to be a mediating factor with respect to the link of attachment problems and the development of BPD [21]. Furthermore, alexithymia also mediates the effect of trauma on altered empathy-for-pain in BPD [31]. Crucially for patients with BPD, alexithymia is strongly associated with self-harm in women [32]. Concerning its clinical relevance, alexithymia has been shown to be prognostically relevant for psychotherapy outcome [3]. Another limiting factor of the present study concerns the lack of emotional challenges during EEG measurement, because it is known that emotional tasks or other stressors may impact on frontal asymmetry [12, 31, 33–35]. This might be worth considering in future research.

## Conclusions

Patients with BPD do not show an aberrant pattern of FEA. However, frontal EEG asymmetry at rest was uniquely correlated with alexithymia in this clinical group. This could suggest that frontal EEG asymmetry may serve as a potential biomarker of clinically relevant psychopathology in BPD.

## Abbreviations

BDI: Beck depression inventory
BPD: Borderline personality disorder
DDF: Difficulties describing feelings
DIF: Difficulties identifying feelings
EEG: Electroencephalography
EOT: Externally oriented thinking
FAS: Frontal EEG asymmetry score
HC: Healthy controls
MDD: Major depressive disorder
MWT-B: Mehrfachwahl–wortschatz–intelligenz test, version B
SCL-90: Symptom checklist of derogatis
TAS-20: Toronto Alexithymia scale
